# Psychiatric Symptoms in Patients with Shiga Toxin-Producing *E. coli* O104:H4 Induced Haemolytic-Uraemic Syndrome

**DOI:** 10.1371/journal.pone.0101839

**Published:** 2014-07-09

**Authors:** Alexandra Kleimann, Sermin Toto, Christian K. Eberlein, Jan T. Kielstein, Stefan Bleich, Helge Frieling, Marcel Sieberer

**Affiliations:** 1 Department of Psychiatry, Social Psychiatry and Psychotherapy, Hannover Medical School, Hannover, Germany; 2 Department of Nephrology and Hypertension, Hannover Medical School, Hannover, Germany; Universitätsklinikum Hamburg-Eppendorf, Germany

## Abstract

**Background:**

In May 2011 an outbreak of Shiga toxin-producing enterohaemorrhagic *E. coli* (STEC) O104:H4 in Northern Germany led to a high number of in-patients, suffering from post-enteritis haemolytic-uraemic syndrome (HUS) and often severe affection of the central nervous system. To our knowledge so far only neurological manifestations have been described systematically in literature.

**Aim:**

To examine psychiatric symptoms over time and search for specific symptom clusters in affected patients.

**Methods:**

31 in-patients suffering from *E. coli* O104:H4 associated HUS, were examined and followed up a week during the acute hospital stay. Psychopathology was assessed by clinical interview based on the AMDP Scale, the Brief Symptom Inventory and the Clinical Global Impressions Scale.

**Results:**

At baseline mental disorder due to known physiological condition (ICD-10 F06.8) was present in 58% of the examined patients. Patients suffered from various manifestations of cognitive impairment (n = 27) and hallucinations (n = 4). Disturbances of affect (n = 28) included severe panic attacks (n = 9). Psychiatric disorder was significantly associated with higher age (p<0.0001), higher levels of C-reactive protein (p<0.05), and positive family history of heart disease (p<0.05). Even within the acute hospital stay with a median follow up of 7 days, symptoms improved markedly over time (p <0.0001).

**Conclusions:**

Aside from severe neurological symptoms the pathology in *E.coli* O104:H4 associated HUS frequently includes particular psychiatric disturbances. Long term follow up has to clarify whether or not these symptoms subside.

## Introduction

From May to July 2011 an outbreak of Shiga toxin-producing enterohaemorrhagic *Escherichia coli* (STEC) O104:H4 infections in Northern Germany occurred. Of the 3.186 reported STEC-patients 845 (22%) presented with haemolytic-uraemic syndrome. Ninety percent of the affected patients were adults [1]. According to the German STEC-HUS registry 69% of all HUS patients presented with neurological symptoms like headache or visual disturbances or neurological signs ranging from tremor and aphasia to delirium, coma and seizures [2]. One explanation for this unusual high incidence of neurological symptoms/signs was the increased virulence of the outbreak-strain O104:H4, which combined virulence factors of typical enteroaggregative and Shiga toxin-producing E. coli and showed an unusual age distribution of the affected patients as STEC-HUS usually occurs in pre-school children, were it shows lower rates of central nervous system (CNS) involvement. [3]

In the majority of cases infection with Shiga toxin-producing *Escherichia coli* leads to watery diarrhoea followed by bloody diarrhoea and abdominal cramps due to colitis [4]. These symptoms can be followed by the haemolytic-uraemic syndrome, which is defined by the sudden onset of microangiopathic haemolytic anaemia, thrombocytopenia and acute kidney injury [5]. In severe cases of HUS patients can also show CNS pathology associated with worse outcome. CNS pathology sometimes occurs before the onset of the haemolytic-uraemic syndrome and shows different manifestations that range from lethargy to seizures and coma [6] [7].

Little is known about the mechanisms causing CNS pathology. In infection with STEC different cytotoxins, Shiga toxin 1 and Shiga toxin 2, play a crucial role in systemic disease. The subunit StxB of Shiga toxin 2 is able to bind to glycolipid receptor, globotriaosylceramide (Gb3), on the cell membrane of target cells. Subunit StxA inhibits protein synthesis within the target cell, leading to its apoptosis [8]. Gb3 expressing cells have been found in the glomerular endothelium and neurons of different animals, in rabbits they determine the localization of pathological lesions [9]. Obata et al localized Gb3 on neurons and vascular endothelial cells within the human CNS [10]. Autopsy of the human CNS showed oedema, hypoxic-ischemic changes, microthrombosis and microhaemorrhages [11]. Former MRI studies in affected humans showed abnormal findings in up to 50% of patients, especially in the basal ganglia, but also in almost every other structure of the central nervous system. These lesions could not be correlated with the variety of clinical symptoms, severity of disorder or outcome, suggesting that the Shiga toxin could also have a direct effect on the central nervous system [3] [12].

In all previous outbreak reports on STEC-HUS specific psychiatric symptoms were not reported. None of the reports used a structured psychiatric evaluation of patients, neither during the acute phase nor during follow up.

The aim of our analysis was to conduct a structured psychiatric evaluation of patients suffering from STEC-HUS amidst the 2011 outbreak in our tertiary care center. Furthermore we were trying to substantiate suspected specific psychiatric symptom clusters suggested by the treating physicians and whenever possible perform a short term follow up during the hospital stay.

## Materials and Methods

### Ethics Statement

This study was performed in accordance with the Declaration of Helsinki.

Data were obtained during psychiatric consultation service and have been analysed retrospectively and anonymously. Results of blood samples, obtained in clinical indication, have been analysed retrospectively as well. The examinations were part of clinical routine treatment and patients gave their verbal informed consent to be examined. Therefore no written informed consent was given by the participants and all data were anonymized and de-identified prior to analysis. All 52 STEC-HUS in-patients treated at the Hannover Medical School participated in a study on the German outbreak of *E. Coli* O104:H4 [2]. Approval for this analysis was obtained from the Ethics Committee of the Hannover Medical School, Permit Number: 1106–2011.

### Study design and patients

During the German outbreak of *E. Coli* O104:H4 from May to July 2011 32 out of 52 STEC- HUS in-patients at our tertiary care hospital received psychiatric consultation service examinations. Systematic psychiatric consultation was initiated after various psychiatric symptoms were noted in many patients by their treating doctors. Due to the lack of data on psychiatric symptoms in infection with *E. Coli* O104:H4 all patients received a structured psychiatric consultation as well as follow up examinations.

One of the 32 screened patients was excluded from the analysis as criteria for HUS were not met. All interviews were conducted by experienced psychiatrists, trained for the assessment of the study. The first interview with the patient was performed after a median of 7 days (range 2 to 23 days) after the onset of diarrhoea. The second interview was performed 7 days later (range 7 to 9 days).

Assessment of baseline data included demographic data like qualification and marital status, psychiatric and medical history, family medical history, present medication, treatment with plasma exchange or dialysis and laboratory results. Afterwards psychopathology was rated using the 115-item Association for Methodology and Documentation in Psychiatry (AMDP) scale. Assessment of global psychological distress and psychopathology with respect to the past seven days was performed using the Brief Symptom Inventory (BSI). Severity of psychiatric symptoms was conducted using the Clinical Global Impressions Scale (CGI). At follow up examination present medication and again psychopathology, severity of psychiatric symptoms and global psychopathological distress was examined using the AMDP scale, the CGI and the BSI (past seven days). Additionally patients' charts, clinical progress documentation and third party informations (physicians, nurses) were collected at baseline and follow up to gain more information about psychopathological abnormalities. 22 Patients completed both assessments, 9 only participated at baseline visit and were discharged before the conduction of the second interview.

### Definition of STEC-HUS and severity of illness

Infection with *E. coli* O104:H4 was confirmed by either positive stool cultures and/or presence of *Shiga toxin*, confirmed either by ELISA or PCR. HUS was defined according to the Robert-Koch-Institute (the national level health authority) definition if at least two out of three criteria were fulfilled: (i) thrombocytopenia (<150×10^9^/L), (ii) microangiopathic haemolytic anaemia lactate dehydrogenase (LDH>240 U/L and haemoglobin <12.0 g/dL), and (iii) acute kidney injury according to the AKIN definition [13] [14].

Severity of illness was assessed through lowest platelet count, highest peak of C-reactive Protein (CRP) and haemolytic activity, which was defined through highest peak of lactate dehydrogenase (LDH) and minimum haemoglobin level.

### Psychiatric Assessment

#### AMDP scale

The AMDP scale was developed in Europe by the Association for Methodology and Documentation in Psychiatry (AMDP) in order to standardize the assessment of psychopathological symptoms [15]. It is based on a semi-structured interview method and consists of 115 items. Each psychiatric symptom is scored from 0 (not at all), one (mild), two (moderate) to three (severe). The AMDP system is the most commonly used and best known psychiatric documentation system in the German-speaking area and has been translated into many other languages [16]. Several studies reported that it has moderate to high interrater reliability for most symptoms and that it can be considered a well-established test with good to very good reliability and validity [17].

#### Brief Symptom Inventory

The BSI was derived from the Symptom Check-List-90-revised (SCL-90-R) and has been used extensively in diverse populations as a screening instrument for global psychological distress [18]. It is a 53-item self-administrated questionnaire that uses five-point Likert scales and measures nine dimensions: somatisation (SOM), obsessive-compulsive (O-C), depression (DEP), anxiety (ANX), interpersonal sensitivity (I-S), hostility (HOS), phobic anxiety (PHO), paranoid ideation (PAR), and psychoticism (PSY). Items of the BSI are assessed using a 5-point scale ranging from ‘not at all’ (0) to ‘extremely’ (4). Patients rate their symptoms with respect to the last seven days. In addition, the inventory comprises three general distress measures: The General Severity Index (GSI) quantifies severity of illness and is used for outcome measurements. The Positive Symptom Distress Index (PSDI) is designed to measure the intensity of symptoms and the Positive Symptom Total (PST) reports the number of symptoms.

Scores are interpreted by comparison to age-appropriate norms. Normative data are available for both clinical and non-clinical samples of adolescents (over 13 years) and adults. The BSI demonstrated strong internal consistency reliability and a strong correlation with the SCL-90-R [19] [20]. Convergent validity was examined in various studies. Studies also showed a good reliability for the German version of the BSI which was used in this study [21].

#### Clinical Global Impressions scale

The Clinical Global Impressions scale is commonly used to measure symptom severity (CGI-S), improvement (CGI-I) and efficacy of treatments in treatment studies of patients with mental disorders [22]. It is based on external rating. The severity of mental illness is rated on the following seven-point scale: 1 = normal, not at all ill; 2 = borderline mentally ill; 3 = mildly ill; 4 moderately ill; 5 markedly ill; 6 severely ill; 7 among the most extremely ill patients. Improvement, in this study assessed after one week, also can be rated on a seven-point scale: 1 = very much improved; 2 = much improved; 3 = minimally improved; 4 = no change; 5 = minimally worse; 6 = much worse; 7 = very much worse. In this observational study the Clinical Global Impression-Efficacy Index has not been used.

### Statistical analysis

Deviation from normal distribution was tested using the Kolmogorov–Smirnov test. Correlation analysis between continuous data was then performed using Pearson's test. Between group comparisons (presence of psychiatric disorder vs. healthy) were performed using t-tests for continuous data and Chi squared or Fisher's exact test for categorical data. For not normally distributed data non-parametric Mann-Whitney U-test was used.

BSI data were compared with a healthy control group (German reference population). For comparement scores from all scales were transformed into t-values (mean = 50; SD = 10). These were calculated according to age and gender specific German norm groups. Between group analyses were performed using two way Analysis of Variance (ANOVA) and Bonferroni's *post-hoc* test when appropriate. Considering the significant correlation between age and presence of psychiatric disorder (p<0.0001) a receiver operating characteristic (ROC) - analysis and calculation of Youden's Index (Sensitivity + Specificity – 1) has been performed.

Results are presented as means (SD). P-values of less than 0.05 (two-tailed) were considered significant. Analysis was performed using the Statistical Package for the Social Sciences (SPSS™) for Windows 16.0.2 (SPSSInc., Chicago,IL) and most data are presented using Graph Pad Prism 5 (Graph Pad Inc., San Diego, CA).

## Results

### Sample characteristics

All 31 patients at baseline and 22 of them at follow up have been included in analysis. The following data describe the sample characteristics at baseline: Women comprised 71% of the sample. Median age was 40 years (SD 19.2) and ranged from 18 to 81 years. Median duration of diarrhoea was 7 days (SD 5) and of associated HUS 8 days (SD 4.5).

Assessed previous illnesses included prior renal disease (n = 0), smoking (n = 1; type 2), diabetes (n = 1), hyperlipidemia (n = 2) and elevated arterial blood pressure (n = 5). Only one patient reported a history of psychiatric disorder (Panic disorder).

### Frequency and characterization of psychiatric symptoms

Table 1 shows frequency of psychopathological abnormalities on the AMDP scale at baseline and follow up. Aggregation of collected data and clinical psychiatric examination revealed that an organic psychiatric disorder (ICD-10 F06.8) was confirmed in 58% of patients. On the CGI only 7 patients (22.6%) were rated as not psychiatric ill, meaning that even more patients suffered from psychopathological abnormalities, though diagnosis criteria for a psychiatric disorder were not fulfilled. At baseline 88% of patients showed disturbances of affect. Mostly feelings of anxiety and panic attacks were reported. Some patients suffered from 10 panic attacks daily. Anxiety was often reported as generalized without obvious specific causes. The second most observed abnormalities were disturbances of attention and memory (88%). These mostly included concentration and attention deficits which were associated with slow and stiff thinking (formal disorders of thought: 78.2%). Four patients suffered from vivid hallucinations. As an illustrating example a patient reported he was seeing different people in his room and described the surrounding as if he was travelling in a train. He reported knowing that these scenic hallucinations could not be real and memorized them in detail after recovery. No patient reported or showed signs of suicidal tendencies.

**Table 1:** Frequency of abnormalities within the AMDP-Scale and other psychiatric findings.

Psychiatric Symptom	Baseline (%)	Follow up (%)
Disorders of consciousness	11 (35.9)	4 (18.2)
Disturbances of orientation	12 (39.1)	5 (22.7)
Disturbances of attention and memory	27 (88)	18 (81.9)
Dyscalculia	6 (19.4)	1 (4.5)
Formal disorders of thought	24 (78.2)	17 (77.3)
Phobias and Compulsions	13 (42.4)	7 (31.9)
Delusions	6 (19.4)	2 (9.1)
Disorders of perception	8 (26)	4 (18.2)
Disorders of ego	5 (16.3)	4 (18.2)
Disturbances of affect	27 (88)	17 (77.4)
Panic attacks	9 (29.0)	9 (40.9)
Disorders of drive and psychomotility	26 (84.8)	10 (45.5)
Circadian disturbances	3 (9.7)	5 (22.7)
Other disturbances	19 (61.3)	11 (50.0)
Organic psychiatric disorder (F06.8)	18 (58.1)	9 (40.9)

### Brief Symptom Inventory

**Figure 1:**
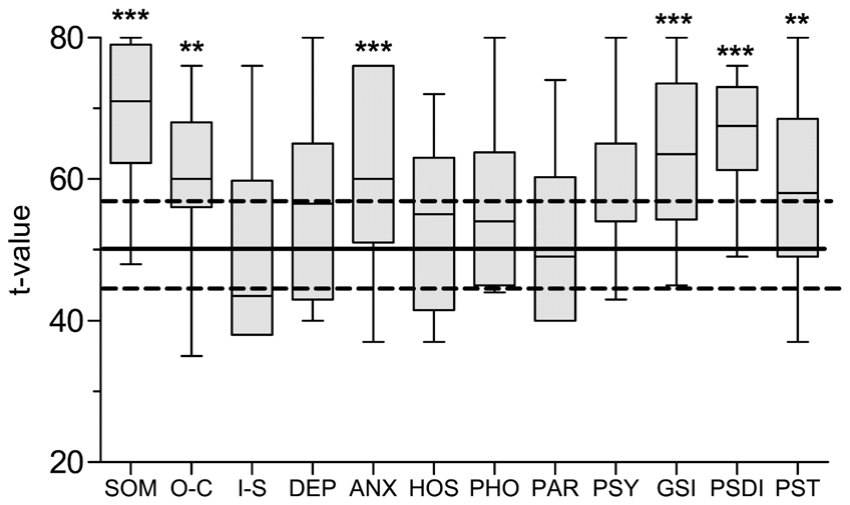
BSI t-values at baseline. Figure 1 shows the results of the Brief Symptom Inventory at baseline as boxplots. Results are compared to a healthy cohort which is marked by horizontal lines (t = 50, SD  = 10). The horizontal line inside the “box” represents the median. The boundaries of the “box” indicate the 25th and 75th percentiles; the whiskers indicate the largest and smallest observed values. P-values given in the figure are derived from Bonferroni's *post hoc* test after two way ANOVA (cases vs controls): **P<0.01 ***P<0.001.

To confirm the findings in the AMDP-Scale in a self-reported instrument, patients completed the BSI. As some patients could not read due to visual disturbances or poor concentration in a few cases questions were read to them. Fig. 1 shows the results of the two way ANOVA analysis comparing patients with an age and gender matched healthy control group (t = 50). Post-hoc analyses show that the clinical sample significantly differs from the healthy controls.

### Laboratory results

There was no statistically significant difference in the levels of lowest platelet count or minimum haemoglobin between healthy and psychiatric affected patients. Presence of psychiatric disorder at baseline and follow-up was significantly associated with higher peak CRP (p<0.05). Presence of psychiatric disorder at follow up was also associated with higher levels of peak LDH (p<0.05).

### Other correlations and significant findings

Psychopathological abnormalities started between 0 to 10 days after onset of HUS (median 1.5 d, SD 2.9), 50% of patients developed symptoms within 24 h of HUS onset.

Table 2 and 3 provide details of the sample characteristics and their correlation with the presence of psychiatric disorder. Fisher's test showed statistically significant results according to age (p<0.0001) and family history of heart disease (p<0.05). ROC-analysis and calculation of Youdens's Index for age younger/older than 30.5 years is shown in Table 4. The observed significance with higher age was also found investigating single items like anxiety (p<0.0001), panic attacks (p<0.005) or slow thinking (p<0.0001).

**Table 2:** Sample characteristics and differences between patients with or without organic psychiatric disorder at baseline.

Characteristics, mean (SD)	No diagnosis of organic psychiatric disorder	F06.8	p-value
Age, years	22 (13.4)	47 (15.3)	<0.0001
Women, n (%)	8 (61.5)	14 (77.8)	0.326
Hight, cm	170 (8)	172 (7)	0.951
Weight, kg	68 (12.2)	75 (14.3)	0.118
Duration of Diarrhoea, days	6 (5.1)	7 (5)	0.630
Duration of HUS, days	8 (2.9)	8 (5)	0.371
Duration of Dialysis, days	4.5 (4.3)	6 (5.2)	0.527

P-values are from Student's T-test except for gender, which is from Chi-squared test.

**Table 3:** Family history of illness and risk factors in patients with or without organic psychiatric disorder.

Characteristics	No diagnosis of organic psychiatric disorder (%)	F06.8 (%)	p-value
Hypertension	0 (0.0)	5 (27.8)	0.058
Family history of …			
Mental disorders	5 (38.5)	6 (33.3)	0.768
Drug addiction	3 (23.1)	3 (16.7)	0.656
Heart disease	3 (23.1)	11 (61.1)	< 0.05
Cancer	7 (53.8)	9 (50)	0.961

P-values are from Chi-squared test, except for high blood pressure and heart disease, which are from Fisher's exact test. Results for other risk factors (diabetes, hyperlipidaemia, nephrological illness) are not shown due to small amount.

**Table 4:** Age and organic psychiatric disorder.

Age	No diagnosis of organic psychiatric disorder	F06.8	p-value (Fisher's test)
< 30 years	12	1	
			<0.0001
> 30 years	1	17	

Dichotomized age groups show a high predictive value for presence of organic psychiatric disorder. Youden's index value is 1.87.

Statistical analysis showed no significant difference between treatment with different types of medication like antibiotics (n = 6), eculizumab (n = 12), anticoagulants (n = 24) or antiepileptics (n = 19) and severity, outcome or presence of psychiatric disorder at baseline or in the completer-group.

### Course of the disease

**Figure 2:**
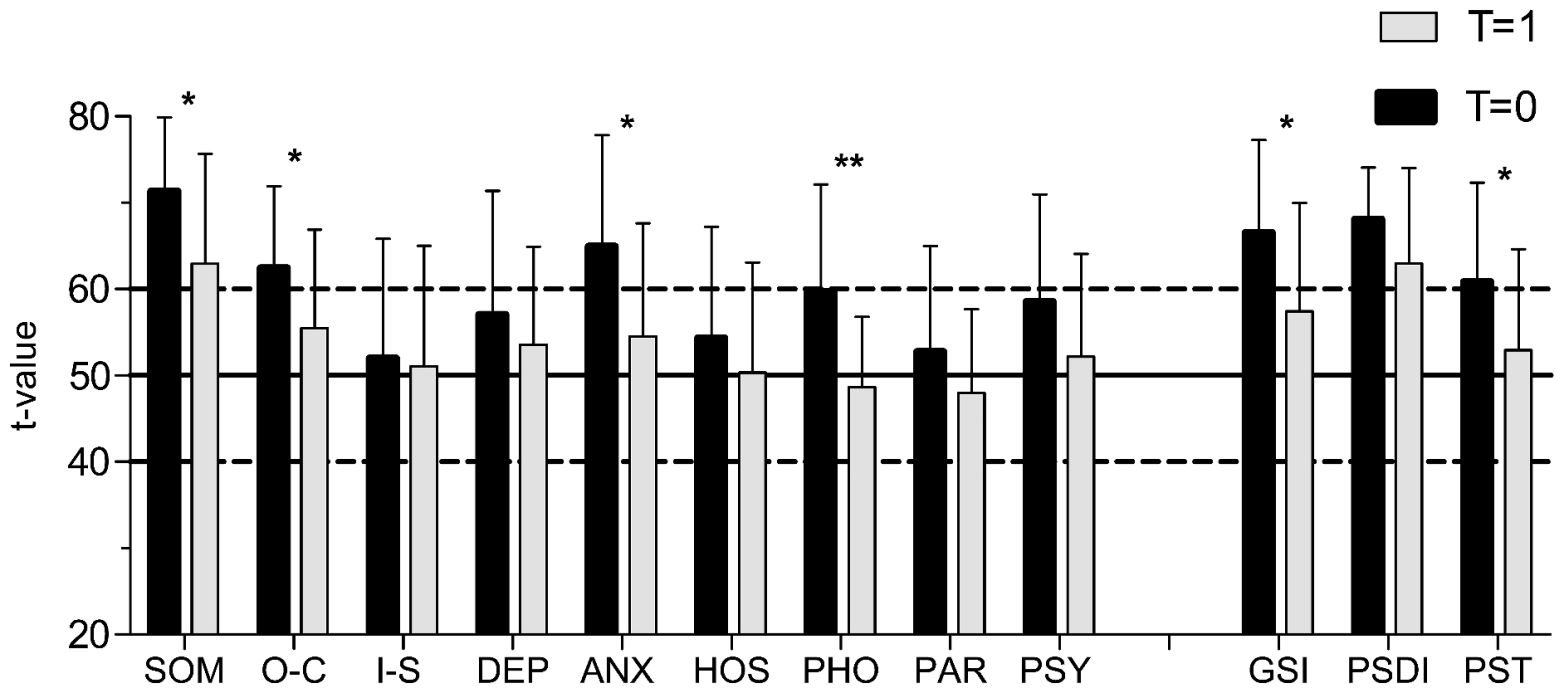
BSI t-values at follow up compared to baseline. BSI t-values of completers at follow up (t = 1) compared to baseline (t = 0). Healthy cohort is marked by horizontal lines (t = 50, SD  = 10). Numbers given in the figure represent mean (SD). P-values given in the figure are derived from Bonferroni's *post hoc* test after two way ANOVA (follow up vs baseline): *P<0.05 **P<0.01.

According to the CGI scale patients significantly improved over time (p<0.0001). Evaluation of the CGI revealed that at follow up 50% of patients scored 1, meaning “much improved”. In only one patient the condition remained unchanged and none showed signs of worsening. However, 9 patients remained with the diagnosis of an organic psychiatric disorder. Fig. 2 shows the *post hoc* analysis of the BSI with all completers between baseline and follow-up.

## Discussion

To our knowledge, we here report the first systematic psychometric analysis of CNS involvement in adult patients with STEC-HUS. Thirty-one in-patients with *E.coli* O104:H4 STEC-HUS during the 2011 German outbreak were studied. The majority of patients (58%) met the ICD-10 criteria for a mental disorder due to a known physical condition. Given that nine patients were already discharged at the follow up exam the frequency of psychiatric symptoms/diagnosis could be even higher.

Presence of psychiatric disorder at baseline was related to age or family history of heart disease, as well as higher CRP levels. CRP levels are known to be increased in patients suffering from depression or anxiety disorders [23]. The strong bidirectional connection between cardiovascular diseases and depression has been examined in various studies, but there are no previous data on susceptibility for psychiatric disorders concerning family history of heart disease [24].

Interestingly neither laboratory and other demographic data than age, nor the prescribed medication were related to the psychiatric symptoms.

The rapid onset of psychiatric symptoms, which occurred within 24 h after diagnosis of HUS in half of the patients, as well as their improvement over time correspond with recently published data from neurological examinations [25]. Our observation that higher age could be a risk factor for development of psychiatric symptoms is in line with a reported significance between higher age and a low score in the Mini-Mental State Examination of affected patients with HUS [26]. Interestingly, this association was not found for other neurological deficits but is stable in our exact analysis of different symptoms (anxiety only, panic attacks only etc.). From the view of a toxin-mediated hypothesis of CNS involvement in STEC-HUS older patients could be more often affected due to a higher vulnerability or weakening of the blood-brain barrier [27]. The ability of Shiga toxin to cross the blood-brain barrier has already been shown in the rat [28]. A recent publication focussing on the neurologic examination reported an elevated albumin ratio in the cerebrospinal fluid (CSF) of four out of five STEC-HUS affected patients. The authors interpreted these findings as a possible sign of blood-CSF barrier dysfunction [29]. Additionally age was also reported to be the overriding risk factor for death in the German STEC-HUS registry [30]. The significant correlation of psychiatric disorder in patients with higher peak CRP but not with lowest haemoglobin or lowest platelet count contributes to the thesis that CRP could be a better fitting laboratory marker of CNS complications in STEC-HUS then the usual indicators for the degree of progression in HUS [31]. Also, an interesting recently published laboratory finding is the alteration of circulating microRNAs (mi-R 126) in STEC-HUS patients with moderate to severe CNS involvement compared to patients with HUS only [32].

The majority of affected patients suffered from anxiety symptoms and/or cognitive dysfunction. At first sight the anxiety syndrome of affected patients could be interpreted as a reaction due to suffering from a life-threatening epidemic disease. Clinical interview on the other hand revealed that most patients were not able to indicate the reason of their anxiety. Anxiety was described as a generalized state of affect, mixed with free floating anxiety and panic attacks up to 10 times daily. The mixture of generalized anxiety and panic attacks is typical for organic anxiety syndromes (ICD-10 F06.4) [33]. Interestingly, some of the patients suffering from panic attacks also reported recurring chest pain. The phobic scale within the BSI could represent sickness behavior and impairment due to diarrhoea and HUS. Specific phobias were negated.

Dysfunction of memory and disturbances of thought mainly included slow thinking and poor concentration. For instance, some patients were not able to read more than a few sentences. Seven patients reported severe dyscalculia.

In contrast to the various neurological findings in CNS pathology, observed psychiatric disturbances were relatively specific. Still, making a precise distinction between neurological and psychiatric symptoms is complicated as both are a sign of CNS affection and long term observations could help in making a more precise distinction.

Focussing on the psychiatric part, the question if a special circuit or anatomical structure is affected predominantly should be considered. Tironi-Farinati et al. showed that intraventricular injection of Shiga toxin 2 causes dendritic abnormalities in rat brains and increases the expression of Gb3 [34]. They also reported a localization of Gb3 expressing cells in the CA1 region of the hippocampus and the striatum of rats. Another recent study conducted by Meuth et al. showed neuronal expression of Gb3 and dose dependent neuronal cytotoxity of Shiga toxin 2 on thalamic neurons and astrocytes of female rats, fitting to recently published research showing symmetric MRI alterations in the lateral thalamus and brainstem (50%) of affected patients [7] [35].

Involvement of the hippocampus and/or thalamus could possibly explain anxiety, panic attacks and disturbances of memory and attention [36] [37]. These questions remain unanswered, as our report only allows a description of psychiatric symptoms, but is not suitable to establish causality.

Beside the possible scientific value of accurate psychiatric examinations, these findings could also be clinically important, as only few patients received a sufficient symptomatic treatment such as benzodiazepines to lower severe anxiety. Though the course of the disease seems to be benign, due to lack of long-term data we cannot exclude the possibility of vulnerable patients developing disorders afterwards. In our outpatient clinic two patients asked for consultancy weeks after discharge.

### Limitations

First this study is limited by its open design and the single center setting, both limitations owed to the acute onset of the crisis making a proper planned randomized multi-center evaluation impossible. Given that the so far largest report on STEC-HUS in adults before the described outbreak involved 22 patients the assessment of 31 patients is rather high [38]. Still, the sample size is small and does not allow interpretations beyond a descriptive level. It is possible that moderate effects of medication or correlation with more laboratory findings only gain statistical significance in a larger cohort.

Second a major limitation of this study is the lack of long-term follow up data and its retrospective design. Though our analysis showed healing tendency for psychiatric disorders it is possible that patients suffer from residual symptoms or new psychiatric disturbances. Thus, there is a great need for further studies on long-term outcome. Third, continuous observation could reveal more data about correlations with other systemical abnormalities, especially neurological deficits, impact on outcome and more specific laboratory results.

### Implications

CNS involvement in patients with *Escherichia coli* 0104:H4 associated haemolytic uraemic syndrome shows high rates of psychiatric affection with a probably benign course of psychiatric disorder. A higher sensitivity for psychiatric disturbances could be important for an optimized disease severity based treatment approach of *Escherichia coli* 0104:H4- associated haemolytic uraemic syndrome. Further studies are needed to assess potential long-term sequelae of STEC-HUS and investigate the underlying mechanisms of these symptoms.

## References

[1] Frank C, Werber D, Cramer JP, Askar M, Faber M, et al (2011) Epidemic profile of shiga-toxin producing Escherichia coli O104:H4 outbreak in Germany. N Engl J Med 365: 1771–1780. 21696328 10.1056/NEJMoa1106483

[2] Kielstein JT, Beutel G, Fleig S, Steinhoff J, Meyer TN, et al (2012) Best supportive care and therapeutic plasma exchange with or without eculizumab in shiga-toxin-producing E. coli O104:H4 induced haemolytic-uraemic syndrome: An analysis of the German STEC-HUS registry. Nephrol Dial Transplant 27: 3807–3815. 23114903 10.1093/ndt/gfs394

[3] Nathanson S, Kwon T, Elmaleh M, Charbit M, Launay EA, et al (2010) Acute neurological involvement in diarrhea-associated hemolytic uremic syndrome. Clin J Am Soc Nephrol 5: 1218–1228. 20498239 10.2215/CJN.08921209 PMC2893076

[4] Andreoli SP, Trachtman H, Acheson DW, Siegler RL, Obrig TG (2002) Hemolytic uremic syndrome: Epidemiology, pathophysiology, and therapy. Pediatr Nephrol 17: 293–298. 11956886 10.1007/s00467-001-0783-0

[5] Proulx F, Seidman EG, Karpman D (2001) Pathogenesis of shiga toxin-associated hemolytic uremic syndrome. Pediatr Res 50: 163–171. 11477199 10.1203/00006450-200108000-00002

[6] Hamano S, Nakanishi Y, Nara T, Seki T, Ohtani T, et al (1993) Neurological manifestations of hemorrhagic colitis in the outbreak of Escherichia coli O157:H7 infection in Japan. Acta Paediatr 82: 454–458. 8518521 10.1111/j.1651-2227.1993.tb12721.x

[7] Magnus T, Rother J, Simova O, Meier-Cillien M, Repenthin J, et al (2012) The neurological syndrome in adults during the 2011 northern German E. coli serotype O104:H4 outbreak. Brain 135: 1850–1859. 22539260 10.1093/brain/aws090

[8] Mallard F, Antony C, Tenza D, Salamero J, Goud B, et al (1998) Direct pathway from early/recycling endosomes to the golgi apparatus revealed through the study of shiga toxin B-fragment transport. J Cell Biol 143: 973–990. 9817755 10.1083/jcb.143.4.973 PMC2132951

[9] Zoja C, Corna D, Farina C, Sacchi G, Lingwood C, et al (1992) Verotoxin glycolipid receptors determine the localization of microangiopathic process in rabbits given verotoxin-1. J Lab Clin Med 120: 229–238. 1323633

[10] Obata F, Tohyama K, Bonev AD, Kolling GL, Keepers TR, et al (2008) Shiga toxin 2 affects the central nervous system through receptor globotriaosylceramide localized to neurons. J Infect Dis 198: 1398–1406. 18754742 10.1086/591911 PMC2684825

[11] Siegler RL, Pavia AT, Christofferson RD, Milligan MK (1994) A 20-year population-based study of postdiarrheal hemolytic uremic syndrome in Utah. Pediatrics 94: 35–40. 8008534

[12] Donnerstag F, Ding X, Pape L, Bultmann E, Lucke T, et al (2012) Patterns in early diffusion-weighted MRI in children with haemolytic uraemic syndrome and CNS involvement. Eur Radiol 22: 506–513. 21979865 10.1007/s00330-011-2286-0

[13] Robert-Koch-Institut (2011) Ausbruchs-Falldefinition für EHEC- und HUS-fälle im Rahmen des Ausbruchs im Frühjahr 2011 in Deutschland. 2013.

[14] Mehta RL, Kellum JA, Shah SV, Molitoris BA, Ronco C, et al (2007) Acute kidney injury network: Report of an initiative to improve outcomes in acute kidney injury. Crit Care 11: R31. 17331245 10.1186/cc5713 PMC2206446

[15] Pietzcker A, Gebhardt R, Strauss A, Stockel M, Langer C, et al (1983) The syndrome scales in the AMDP-system. Mod Probl Pharmacopsychiatry 20: 88–99. 6621559 10.1159/000407832

[16] Moller HJ (2009) Standardised rating scales in psychiatry: Methodological basis, their possibilities and limitations and descriptions of important rating scales. World J Biol Psychiatry 10: 6–26. 18663668 10.1080/15622970802264606

[17] Renfordt E, Busch H, von Cranach M, Gulbinat W, Tegeler J (1983) Particular aspects of the interrater reliability of the AMDP psychopathology scale. Mod Probl Pharmacopsychiatry 20: 125–142. 6621547 10.1159/000407836

[18] Derogatis LR, Melisaratos N (1983) The brief symptom inventory: An introductory report. Psychol Med 13: 595–605. 6622612

[19] Piersma HL, Reaume WM, Boes JL (1994) The brief symptom inventory (BSI) as an outcome measure for adult psychiatric inpatients. J Clin Psychol 50: 555–563. 7983203 10.1002/1097-4679(199407)50:4<555::aid-jclp2270500410>3.0.co;2-g

[20] Recklitis CJ, Rodriguez P (2007) Screening childhood cancer survivors with the brief symptom inventory-18: Classification agreement with the symptom checklist-90-revised. Psychooncology 16: 429–436. 16929465 10.1002/pon.1069

[21] Franke H (2000) BSI. Brief Symptom Inventory - deutsche Version. Manual. Göttingen: Beltz.

[22] Guy W (1976) Clinical global impressions. ECDEU Assessment Manual for Psychopharmacology National Institute of Mental Health, Rockville, MD; revised

[23] Duivis HE, Vogelzangs N, Kupper N, de Jonge P, Penninx BW (2010) Differential association of somatic and cognitive symptoms of depression and anxiety with inflammation: findings from the Netherlands Study of Depression and Anxiety (NESDA). J Am Coll Cardiol 29;56(1): 31–7. 10.1016/j.psyneuen.2013.01.002 23399050

[24] Nemeroff CB, Goldschmidt-Clermont PJ (2012) Heartache and heartbreak—the link between depression and cardiovascular disease. Nat Rev Cardiol 9(9): 526–39. 22733213 10.1038/nrcardio.2012.91

[25] Greinacher A, Friesecke S, Abel P, Dressel A, Stracke S, et al (2011) Treatment of severe neurological deficits with IgG depletion through immunoadsorption in patients with Escherichia coli O104:H4-associated haemolytic uraemic syndrome: A prospective trial. Lancet 378: 1166–1173. 21890192 10.1016/S0140-6736(11)61253-1

[26] Weissenborn K, Donnerstag F, Kielstein JT, Heeren M, Worthmann H, et al (2012) Neurologic manifestations of E coli infection-induced hemolytic-uremic syndrome in adults. Neurology 79: 1466–1473. 22993286 10.1212/WNL.0b013e31826d5f26

[27] Dankbaar JW, Hom J, Schneider T, Cheng SC, Lau BC, et al (2009) Age- and anatomy-related values of blood-brain barrier permeability measured by perfusion-CT in non-stroke patients. J Neuroradiol 36: 219–227. 19251320 10.1016/j.neurad.2009.01.001

[28] Lucero MS, Mirarchi F, Goldstein J, Silberstein C (2012) Intraperitoneal administration of shiga toxin 2 induced neuronal alterations and reduced the expression levels of aquaporin 1 and aquaporin 4 in rat brain. Microb Pathog 53: 87–94. 22610042 10.1016/j.micpath.2012.05.005

[29] Skripuletz T, Wurster U, Worthmann H, Heeren M, Schuppner R, et al. (2013) Blood-cerebrospinal fluid barrier dysfunction in patients with neurological symptoms during the 2011 northern German E. coli serotype O104:H4 outbreak. Brain. 10.1093/brain/aws361 23404332

[30] German EHEC-HUS Registry (2011) The German 2011 epidemic of shiga toxin-producing E. coli—the nephrological view. Nephrol Dial Transplant 26: 2723–2726. 21852273 10.1093/ndt/gfr462

[31] Teramoto T, Fukao T, Hirayama K, Asano T, Aoki Y, et al (2009) Escherichia coli O-157-induced hemolytic uremic syndrome: Usefulness of SCWP score for the prediction of neurological complication. Pediatr Int 51: 107–109. 19371288 10.1111/j.1442-200X.2008.02672.x

[32] Lorenzen JM, Menne J, Schmidt BM, Schmidt M, Martino F, et al (2012) Circulating microRNAs in patients with shiga-toxin-producing E. coli O104:H4 induced hemolytic uremic syndrome. PLoS One 7: e47215. 23071762 10.1371/journal.pone.0047215 PMC3469502

[33] World Health Organization. World health organization: The ICD-10 classification of mental and behavioral disorders. Clinical descriptions and diagnostic guidelines. Geneva, world health organization, 1992

[34] Tironi-Farinati C, Loidl CF, Boccoli J, Parma Y, Fernandez-Miyakawa ME, et al (2010) Intracerebroventricular shiga toxin 2 increases the expression of its receptor globotriaosylceramide and causes dendritic abnormalities. J Neuroimmunol 222: 48–61. 20347160 10.1016/j.jneuroim.2010.03.001

[35] Meuth SG, Gobel K, Kanyshkova T, Ehling P, Ritter MA, et al (2012) Thalamic involvement in patients with neurologic impairment due to shiga toxin 2. Ann Neurol. 10.1002/ana.23814 23424019

[36] McGaugh JL (2000) Memory—a century of consolidation. Science 287: 248–251. 10634773 10.1126/science.287.5451.248

[37] Gross CT, Canteras NS (2012) The many paths to fear. Nat Rev Neurosci 13: 651–658. 22850830 10.1038/nrn3301

[38] Dundas S, Murphy J, Soutar RL, Jones GA, Hutchinson SJ, et al (1999) Effectiveness of therapeutic plasma exchange in the 1996 Lanarkshire escherichia coli O157:H7 outbreak. Lancet 354: 1327–1330. 10533860 10.1016/s0140-6736(99)01251-9

